# Metformin inhibits histone H2B monoubiquitination and downstream gene transcription in human breast cancer cells

**DOI:** 10.3892/ol.2014.2158

**Published:** 2014-05-19

**Authors:** YU DU, HAIYAN ZHENG, JIANG WANG, YE REN, MI LI, CHEN GONG, FEI XU, CAIHONG YANG

**Affiliations:** 1 Department of Orthopedics, Tongji Hospital, Huazhong University of Science and Technology, Wuhan, Hubei 430030, P.R. China; 2 Department of Rheumatology, Wuhan Integrated TCM and Western Medicine Hospital (Wuhan No. 1 Hospital), Tongji Medical College, Huazhong University of Science and Technology, Wuhan, Hubei 430030, P.R. China

**Keywords:** metformin, histone H2B, breast cancer, AMPK

## Abstract

Metformin, one of the most widely prescribed antihyperglycemic drugs, has recently received increasing attention for its potential effects with regard to cancer prevention and treatment. However, the mechanisms behind the suppression of cancer cell growth by metformin remain far from completely understood. The aim of the present study was to investigate whether metformin could regulate histone modification and its downstream gene transcription, and its potential function in inhibiting breast cancer cell proliferation. A T47D cell proliferation curve was determined by cell counting following metformin treatment with differing doses or time courses. The cell cycle was analyzed by flow cytometry with propidium iodide staining. Histone H2B monoubiquitination was evaluated by western blotting subsequent to histone extraction. The histone H2B monoubiquitination downstream gene expression level was determined by quantitative PCR. The results showed that metformin changed the cell-cycle check-point and inhibited breast cancer cell proliferation in a dose-dependent manner. AMPK was activated and histone H2B monoubiquitination and downstream gene transcription were inhibited following metformin treatment in the T47D cells. The effect of metformin on T47D cell proliferation was dependent on AMPK activity. It was concluded that metformin can suppress breast cancer cell growth by the activation of AMPK and the inhibition of histone H2B monoubiquitination and downstream gene transcription. This study reveals a novel potential mechanism of cancer cell growth suppression by metformin.

## Introduction

Metformin is one of the first-line drugs used for type 2 diabetes treatment and has been used for over half a century. Recent studies found that metformin not only effectively reduced hepatic glucose production and increased insulin sensitivity, but that it also was effective in decreasing the risk of cancer in patients with type 2 diabetes, inhibiting the growth of cancer cells and enhancing the effects of chemotherapeutic drugs (1).

The potentially beneficial effects of metformin against cancer are believed to be mediated mainly by 5′-adenosine monophosphate-activated protein kinase (AMPK), a well-conserved energy sensor that plays a key role in the regulation of protein and lipid metabolism in response to changes in fuel availability. Activated AMPK inhibits cell growth and proliferation, and therefore antagonizes cancer cell growth (2).

However, more recent data have indicated that metformin can inhibit proliferation and sensitize cancer cells to anticancer drugs through the inhibition of HO-1, by targeting Raf-ERK-Nrf2 signaling in an AMPK-independent manner. Therefore, the mechanisms of the suppression of cancer cell growth by metformin remain unclear (3).

Previous studies have demonstrated the inhibition of metformin on cancer cells (3–7). In the present study, we aimed to understand the anti-tumor molecular mechanisms of metformin.

## Materials and methods

### Cell line and culture conditions

The human mammary carcinoma T47D cell line was purchased from American Type Culture Collection (Manassas, VA, USA). The T47D cells were maintained with RPMI 1640 (Gibco, Invitrogen Life Technologies, Carlsbad, CA, USA) supplemented with 10% fetal calf serum (Hyclone, Thermo Scientific, Waltham, MA, USA), penicillin (100 U/ml) and streptomycin (100 μg/ml). The cells were cultured at 37°C in 5% CO_2_. Additionally, 0.25% trypsin was purchased from Gibco. Metformin was purchased from Sigma-Aldrich (St. Louis, MO, USA).

### Flow cytometry analysis

The T47D cells were cultured in 6-well plates and treated with 4 mM metformin for 48 h, then fixed by 70% ethyl alcohol, which was subsequently removed by centrifugation at 250 × g for 5 min. RNase A was added in for 30 min. Following propidium iodide staining, the cell cycle was detected with a FACSCalibur flow cytometer (BD Biosciences, San Jose, CA, USA).

### Western blotting analysis

Western blotting was conducted, as previously described (7,8). For histone extraction, the cells were lysed with ice-cold NETN buffer containing 10 mM NaF and 50 mM β-glycerophosphate, and following centrifugation at 250 × g for 5 min, the remaining pellets were washed twice with ice-cold PBS and then treated with 200 μl 0.2 HCl. The supernatants were neutralized with 40 μl 1N NaOH, and the sample was loaded onto 12.5% SDS-PAGE gels for western blotting with the indicated antibodies. Monoclonal rabbit anti-human anti-phospho-acetyl-CoA carboxylase (Ser79) (pACC1), monoclonal rabbit anti-human anti-phospho-AMPKα (Thr172)(p-AMPKα1), monoclonal rabbit anti-human anti-ubiquityl-histone H2B (Lys120) (H2B K120ub) and rabbit monoclonal anti-human anti-AMPKα1 were purchased from Cell Signaling Technology, Inc. (Danvers, MA, USA), with the exception of monoclonal mouse anti-human β-actin, was purchased from Sigma-Aldrich.

### Quantitative (q)PCR

Total mRNA was isolated from the cells with TRIzol (Invitrogen Life Technolgies), and then complementary DNA (cDNA) was synthesized from 500 ng total RNA with the PrimeScript^®^ 1st Strand cDNA Synthesis kit (Takara Biotechnology, Dalian, China). qPCR was performed on a 7500RT-PCR System (Applied Biosystems, Foster City, CA, USA) using the SYBR Green detection system with the following program: 95°C for 5 min for 1 cycle, followed by 95°C for 30 sec and 60°C for 45 sec for 40 cycles. The relative expression of the target genes, including p21, cyclin D1, Tulp4 and β-actin, was represented by 2^−ΔΔCT^. All samples were normalized to the β-actin mRNA levels, and the relative expression of the mRNA of every treatment group was calculated. The experiment was duplicated three times. The primer sequences that were used are as follows: Cyclin D1 forward, 5′-ACGCTTCCTCTCCAGAGTGAT-3′ and reverse, 5′-TTGACTCCAGCAGGGCTT-3′; Tulp4 forward, 5′-GGGCCACAATAGCGAGGTT-3′ and reverse, 5′-CCACACGAATATGCCTCCGT-3′; p21 forward, 5′-TGTCCGTCAGAACCCATGC-3′ and reverse, 5′-AAAGTCGAAGTTCCATCGCTC-3′; and β-actin forward, 5′-GTCTGCCTTGGTAGTGGATAATG-3′ and reverse, 5′-TCGAGGACGCCCTATCATGG-3′.

### Cell proliferation

The CellTiter-Blue assay kit (Promega, Southampton, UK) was used to measure the number of cells, according to the manufacturer’s instructions. Briefly, in a 96-well plate, the cells were washed three times with PBS and 20 μl of CellTiter-Blue reagent (Promega) was added. The plate was incubated for 4 h protected from light, and the fluorescence intensity was recorded (excitation, 560 nm; emission, 590 nm) on a Tecan M200 microplate reader (Tecan Australia, Port Melbourne, Vic, Australia).

### Small interfering (si)RNA transfections

siRNA targeting AMPKα1 and a siRNA transfection reagent were obtained from Santa Cruz Biotechnology, Inc. (Santa Cruz, CA, USA). The T47D cells, grown to 50% confluence, were transfected with AMPKα1siRNA or a non-specific control siRNA. After 48 h, the cells were used for experimentation.

### Statistical analysis

The results are presented as the mean ± standard deviation. The statistical analysis was performed with SPSS 13.0 software (SPSS, Inc., Chicago, IL, USA). For comparisons between multiple groups, a one-way analysis of variance was used, while for a comparison between two groups, the SNK method was used. For comparisons between the treatment and control groups, Dunnett’s t-test was used, and for the analysis of differences between groups, the Student’s t-test was used. P<0.05 was considered to indicate a statistically significant difference.

## Results

### Metformin inhibits breast cancer cell proliferation and induces cell cycle arrest

**Figure 1:**
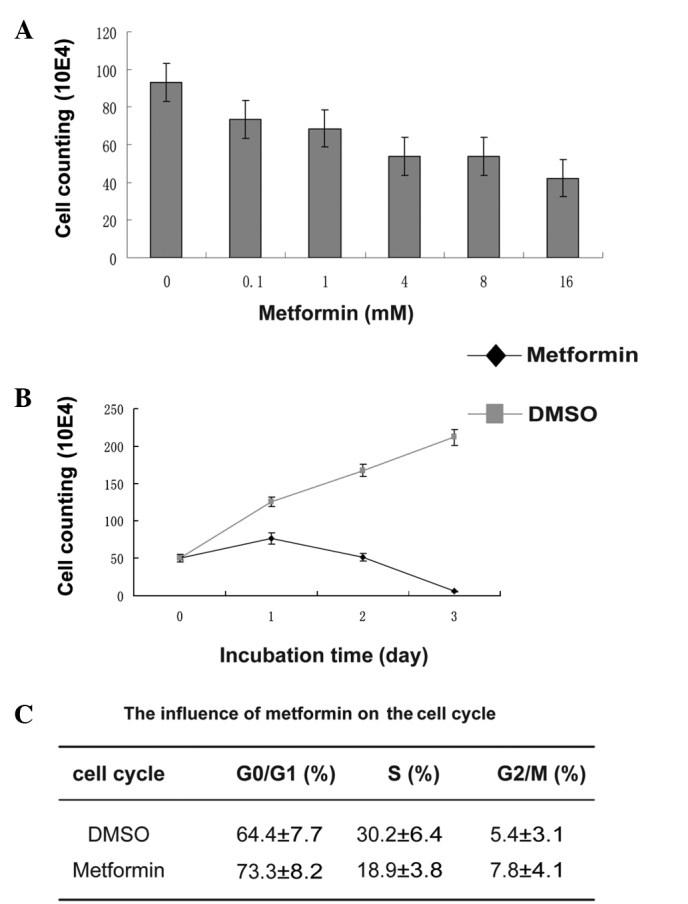
Metformin inhibits T47D cell proliferation and induces cell cycle arrest. (A) The T47D cells were treated with the indicated metformin concentrations, and 12 h later, cell proliferation was determined using Cell Titer-Blue cell counting. (B) The T47D cells were treated with 8 mM metformin for the indicated times, and the viability of these treated cells was determined using Cell Titer-Blue cell counting. (C) The T47D cells were treated with 4 mM metformin for 48 h, then fixed and stained with propidium iodide. The cell cycle was detected by flow cytometry. Data are presented as the mean ± standard deviation (n=3). DMSO, dimethyl sulfoxide.

To detect the effect of metformin on breast cancer cell proliferation, the T47D cells were treated with increasing doses of metformin for 24 h. The inhibition of T47D cell growth by metformin occurred in a dose-dependent manner (Fig. 1A). The cells were almost completely killed by 8 mM metformin for 72 h (Fig. 1B). The cell cycle of the T47D cells was analyzed by flow cytometer following treatment with 4 mM metformin for 48 h; the ratio of the cells in G_0_/G_1_ phase increased from 64.4 to 73.3%, while that of cells in the S-phase dropped from 30.2 to 18.9% (Fig. 1C). These results showed that metformin significantly inhibited the proliferation of the cells and induced cell cycle arrest.

### Metformin activates AMPK and inhibits histone H2B monoubiquitination and downstream gene transcription

**Figure 2:**
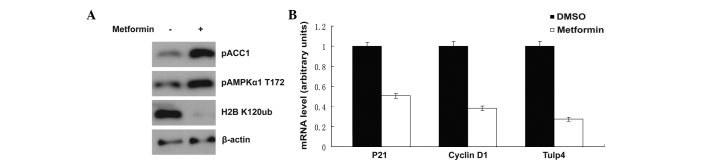
Metformin activates AMPK and suppresses histone H2B monoubiquitination and downstream gene transcription. (A) The T47D cells were treated with 4mM metformin for 12 h, and then the cells were lysed and immnoblotted to determine AMPKα1 T172 phosphorylation and H2B K120 monoubiquitination. β-actin was used as a loading control. (B) The T47D cells were treated with 4mM metformin for 12 h, then mRNA was extracted and the transcription level of p21, cyclin D1 and Tulp4 were examined by quantitative (q)PCR. Data are presented as the mean ± standard deviation (n=3). DMSO, dimethyl sulfoxide; AMPK, 5′-adenosine monophosphate-activated protein kinase.

Metformin is a well-known AMPK activator. Upon treatment with metformin, AMPK was activated, as shown in Fig. 2A, AMPK threonine 172 phosphorylation was increased and the phosphorylation of acetyl-CoA carboxylase at serine 79 was markedly enhanced (Fig. 2A). This result was consistent with the results of previous studies (5–9). Notably, histone H2B monoubiquitination at lysine 120 was inhibited at the same time. It was reported previously that when the cells were suffering a shortage of glucose, the H2B monoubiquitination at lysine 120 was also inhibited (10). The H2B monoubiquitination at lysine 120 was associated with the transcription of multiple downstream target genes (11,12). In the present study, transcription of the downstream genes, including p21, cyclin D1 and Tulp4, was detected by qPCR, and the mRNA level was shown to be decreased significantly following exposure to metformin (Fig. 2B).

### Metformin inhibits breast cancer cell proliferation, which is dependent on AMPK

**Figure 3:**
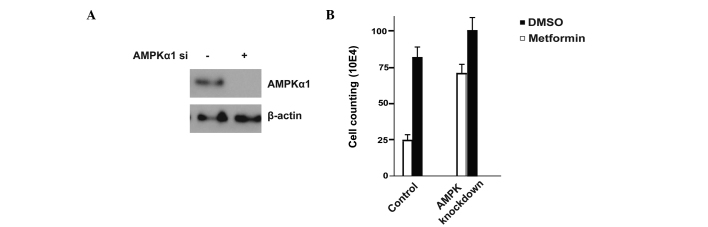
Metformin effect on T47D cell proliferation depends on AMPK. (A) Western blotting showing the AMPKα1 protein level in the T47D cells following transfection with AMPKα1 siRNA. β-actin was used as a loading control. (B) The T47D cells transfected with control siRNA or AMPKα1 siRNA were treated with 4 mM metformin for 12 h, and the cell proliferation was examined by Cell Titer-Blue cell counting. Data are presented as the mean ± standard deviation (n=3). DMSO, dimethyl sulfoxide; AMPK, 5′-adenosine monophosphate-activated protein kinase.

Since metformin efficiently activated the AMPK signal transduction pathway, the study also detected whether the inhibition of cell proliferation by metformin was AMPK-dependent. AMPKα1 protein was specifically knocked down by AMPKα1 siRNA (Fig. 3A). The AMPKα1 siRNA and control groups were then treated with 4 mM metformin for 24 h. The inhibition of T47D cell proliferation by metformin was found to be less effective in the AMPKα1 siRNA group (Fig. 3B). This experiment confirmed that the inhibition of cell proliferation by metformin was AMPK-dependent.

## Discussion

An increasing number of studies are showing that diabetic patients treated with metformin have a lower incidence of cancer compared with those on other treatments (6,13,14). Another large case-control study has indicated that metformin may somewhat reduce the incidence of pancreatic cancer (15). Early-stage clinical trials are currently underway to investigate the potential of metformin to prevent an array of cancers, including colorectal, prostate, endometrial and breast cancer (16–18). However, the underlying mechanism remains to be fully elucidated.

A few of the beneficial effects of metformin have been shown to work through the activation of AMPK. Treatment with metformin results in the activation of AMPK in *in vitro* and *in vivo* experiments, and the activation of AMPK is well known to inhibit the expression of gluconeogenic genes and to promote the expression of enzymes required for fatty acid oxidation (19–21).

However, it has also been reported that in AMPK-knockout cells, metformin works through inhibition of HO-1 by targeting Raf-ERK-Nrf2 signaling, which indicates that a novel mechanism is present (3).

The results of the present study indicated that metformin significantly inhibited the proliferation of the breast cancer cells and induced cell cycle arrest in an AMPK-dependent manner. This is consistent with the results of previous studies. Further molecular mechanism studies showed that metformin activates the AMPK signal transduction pathway, promoting the phosphorylation of ACC1, so that the synthesis of fatty acids of carcinoma cells is inhibited. As a result, the proliferation of the cells was reduced. Unexpectedly, metformin was able to inhibit histone H2B K120-ub, which is related to the transcription of downstream target genes, such as p21 and cyclin D1, which function as regulators of the cell cycle (11,12). The results of qPCR detection indicated that the transcription of p21, Tulp4 and cyclin D1 was inhibited by metformin. This partially explained the mechanism by which metformin blocks the cell cycle.

The present study revealed the possible novel anticancer mechanism of metformin. However, the manner by which metformin inhibits H2B monoubiquitination remains unknown. It is presumed that metformin can activate AMPK, which phosphorylates a certain substrate. This substrate is capable of inhibiting the histone ubiquitination mediated by E3 ubiquitin-protein ligase. Further studies should be performed on the relevant molecular mechanism involved.
